# In vitro analysis of promoter activity in Müller cells

**Published:** 2008-04-23

**Authors:** Scott F. Geller, Phillip S. Ge, Meike Visel, John G. Flannery

**Affiliations:** 1 Helen Wills Neuroscience Institute; 2 Department of Molecular and Cell Biology; 3 Department of Vision Science, University of California, Berkeley, CA

## Abstract

**Purpose:**

Rational modification of promoter architecture is necessary for manipulation of transgene activity and requires accurate deciphering of regulatory control elements. Identification of minimally sized promoters is critical to the design of viral vectors for gene therapy. To this end, we evaluated computational methods for predicting short DNA sequences capable of driving gene expression in Müller cells.

**Methods:**

We measured enhanced green fluorescent protein (eGFP) expression levels driven by “full-length” promoters, and compared these data with computationally identified shorter promoter elements from the same genes. We cloned and screened over 90 sequences from nine Müller cell-associated genes: *CAR2*, *CD44*, *GFAP*, *GLUL*, *PDGFRA*, *RLBP1*, *S100B*, *SLC1A3*, and vimentin (*VIM*). We PCR-amplified the “full-length” promoter (~1500 bp), the proximal promoter (~500 bp), and the most proximal evolutionarily conserved region (ECR; 95–871 bp) for each gene, both with and without their respective 5′ untranslated regions (UTRs), from C57BL/6J mouse genomic DNA. We selected and cloned additional ECRs from more distal genomic regions (both 5′ and 3′) of the *VIM* and *CD44* genes, using both mouse and rat (Sprague-Dawley) genomic DNA as templates. PCR products were cloned into the pFTMGW or pFTM3GW lentiviral transfer vectors. Plasmid constructs were transfected into rat (wMC) or human (MIO-M1) Müller cells, and eGFP expression levels were evaluated by fluorescence microscopy and flow cytometry. Selected constructs were also examined in NIH/3T3 and Neuro-2a cells.

**Results:**

Several ECRs from the nine Müller cell-associated genes were able to drive reporter gene expression as well as their longer counterparts. Preliminary comparisons of ECRs from the *VIM* and *CD44* genes suggested that inclusion of UTRs in promoter constructs resulted in increased transgene expression levels. Systematic comparison of promoter activity from nine Müller cell-expressed genes supported this finding, and characteristic regulation profiles were evident among the different genes tested. Importantly, individual cloned promoter sequences were capable of driving distinct levels of transgene expression, resulting in up to eightfold more cells expressing eGFP with up to 3.8-fold higher mean fluorescence intensity (MFI). Furthermore, combining constructs into single regulatory “units” modulated transgene expression, suggesting that secondary gene sequences provided in *cis* may be used to fine-tune gene expression levels.

**Conclusions:**

In this study, we demonstrate that computational and empirical methods, when used in combination, can efficiently identify short promoters that are active in cultured Müller cells. In addition, the pFTM3GW vector can be used to study the effects of combined promoter elements. We anticipate that these methods will expedite the design and testing of synthetic/chimeric promoter constructs that should be useful for both in vitro and in vivo applications.

## Introduction

Therapeutic treatment of dominant disease in the human retina has yet to be attempted. Given the extreme heterogeneity of retinal diseases (RetNet), effective therapies will likely require more sophisticated vector designs than are currently available for gene augmentation of recessive null mutations. One of the major challenges in engineering any genetic therapy is to design a transgene cassette that enables precise regulatory control of gene expression while abiding by the size constraints of the virus’ packaging limit. Expression of transgenes by targeted cells requires coordination of gene delivery, nuclear localization, and subsequent harnessing of the cell’s transcriptional machinery. Though viral targeting of retinal cells has improved in recent years [1,2], a critical aspect of any gene therapy is how to control the specificity and expression levels of the gene product being expressed [3] once it is delivered to the proper cell.

Progress in achieving precise control over transgene expression is hampered by an incomplete understanding of the underlying genetic mechanisms influencing endogenous gene expression [4]. In recent years, numerous promoters have been characterized and used to confer inducible, constitutive, cell specific, as well as temporal transgene expression to retinal (and many other) cells. However, experimental gene augmentation strategies often utilize strong, constitutive promoters for driving transgene expression in retinal photoreceptors, retinal ganglion cells, and pigmented epithelial cells [5–7]. There is concern that promiscuous, high-expression promoter elements, such as cytomegalovirus (CMV) and chicken beta actin (CBA) [6,7], can generate undesirable and toxic gene expression, particularly when “bioactive” molecules are delivered. Although robust gene expression is often preferable for reporter gene-associated assays used in the laboratory, improved precision and control of gene expression levels will be crucial for human therapies. To this end, novel approaches are being considered for controlling expression levels in diverse cellular contexts [8,9], and synthetic control over viral transgene expression in retinal gene therapy applications is now feasible [10–14].

In addition to imparting improved control overexpression levels, it is critical to identify promoters that are small enough to be efficiently packaged into viral capsids [15]. For example, adeno-associated virus (AAV), which has a relatively small (~4.7 kb) packaging limit [16], continues to be the most widely considered virus for treating eye disease [5,7]. There are currently no reliable methods for predicting how the primary gene sequence and nuclear microenvironment(s) combine to direct, control, and regulate gene expression [17]. Nevertheless, recent advances in computational genomics have provided valuable tools for cross-genome data mining, and have made promoter selection less empirical [18–20]. These online bioinformatics tools generally provide alignments of whole genes as well as chromosomes and allow for rapid identification of evolutionarily conserved regions (ECRs): small stretches of genomic DNA (gDNA) that have survived prolonged selective pressure and presumably contribute to proximate gene expression. These sequences typically contain conserved, empirically annotated transcription factor binding sites, which likely contribute to mRNA expression by influencing both expression levels and cellular specificity [18,21]. Therefore, identification of ECRs from regulatory regions of genes with restricted expression patterns, i.e., in a targeted cell type, is a logical starting point in the search for compact promoters for viral gene therapy.

Previously [22] we modified a self-inactivating lentiviral (LV) transfer vector, pFUGW [23,24], to facilitate the throughput, cloning, and evaluation of novel, computationally identified promoter constructs. In the current study, promoters and ECR promoter fragments from nine Müller cell-associated genes were cloned into our modified vector (pFTM3GW) and studied by transfection and flow cytometry (accession numbers refer to *Mus musculus* genes): carbonic anhydrase II (*CAR2*; NM_009801) [25]; *CD44* (NM_009851) [26]; glial fibrillary acidic protein (*GFAP*; NM_010277) [27]; glutamate-ammonia ligase (*GLUL*; NM_008131; historically referred to as glutamine synthetase, *GS*) [28]; platelet derived growth factor receptor, alpha polypeptide (*PDGFRA*; NM_011058) [29]; retinaldehyde binding protein 1 (*RLBP1*; NM_020599) [30]; S100 protein, beta polypeptide, neural (*S100B*; NM_009115) [31,32]; solute carrier family 1 (glial high affinity glutamate transporter), member 3 (*SLC1A3*; NM_148938; historically referred to as glutamate-aspartate transporter, *GLAST*) [33,34]; and vimentin (*VIM*; NM_011701) [35].

## Methods

### Vector design

**Figure 1:**
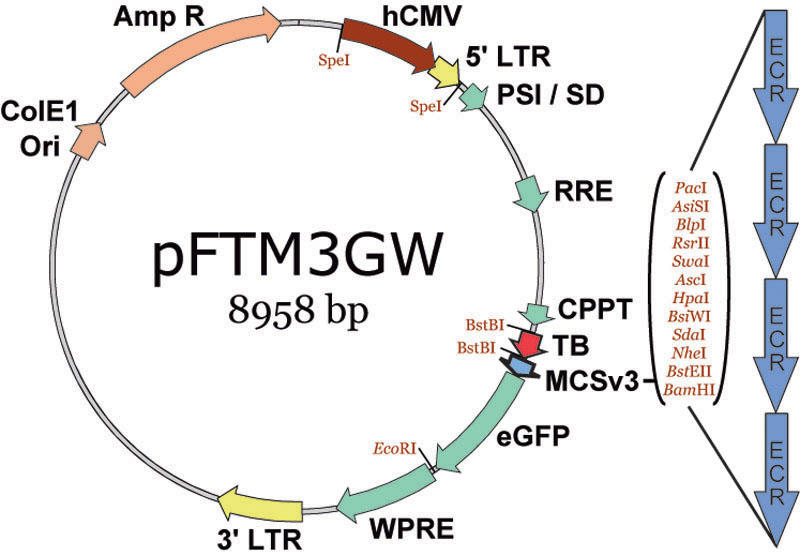
The pFTM3GW lentiviral transfer vector. The pFTM3GW plasmid vector was constructed by adding restriction sites to the parent plasmid, pFTMGW. A SpeI site was added to the 5′ end of the hCMV enhancer, and a BstBI site was added to the 3′ end of the TB; both modifications duplicate existing sites on the opposite sides of the genetic elements, simplifying removal of the elements when desired. We also reordered the restriction sites in the multiple cloning site (MCS) to facilitate double-digestion reaction performance and subsequent cloning. ColE1 ori (bacterial origin of replication) and AmpR (ampicillin resistance gene) were used for plasmid replication. The enhanced green fluorescent protein (eGFP) is the reporter molecule. The cytomegalovirus promoter (CMV), long-terminal repeats (LTRs), splice donor and viral packaging sequences (SD/Psi), Rev Response element (RRE), central polypurine tract (CPPT) and Woodchuck hepatitis virus post-transcriptional regulatory element (WPRE) are viral elements.

We modified a LV transfer vector (pFTMGW) [22] by adding restriction sites and enhancing the multiple cloning site (MCSv3); the new vector was named pFTM3GW (Figure 1). Briefly, we replaced the original multiple cloning site (MCS) in pFTMGW with MCSv3, in which the order of the 12 unique restriction sites was modified (5′-PacI-AsiSI-BlpI-RsrII-SwaI-AscI-*Hpa*I-BsiWI-SdaI-NheI-BstEII-*Bam*HI-3′) to facilitate double-digestion reactions (the BsiWI site was newly added). In addition, we flanked the transcription blocker (TB) with an additional BstBI restriction site, facilitating rapid removal of the TB before LV production. Finally, the hCMV/LTR hybrid promoter was modified such that it was flanked by SpeI endonuclease sites, allowing for efficient removal during preliminary candidate promoter evaluation, if desired.

### Candidate sequence selection

Using online bioinformatics software (dcode) we identified and cloned full-length promoters (~1500 bp), proximal promoter regions (500 bp), and the most proximal ECRs, relative to the transcription start site (TSS), for each of nine Müller cell expressed genes: *CAR2*, *CD44*, *GFAP*, *GLUL*, *PDGFRA*, *RLBP1*, *S100B*, *SLC1A3*, and *VIM*. ECR sequences were identified in canonical promoter regions mandating a minimum of 70% sequence homology between human and mouse genomes over a 100 bp “window.” Based on the observation that several genes have sizable 5′ untranslated regions (UTRs) containing numerous transcription factor binding sites, in addition to the fact that 5′ UTRs afforded improved expression in first-generation constructs, fragments from each of the nine genes (1500 bp, 500 bp, and ECR) were cloned with and without their associated 5′ UTR, or part thereof (see Appendix 1). All transcription start sites were identified using RefSeq sequences (NCBI) compiled into single data files available at (ECRbase). The current mouse (mm8), rat (rn4), and human (hg18) assemblies of their respective genomes were used for all sequence comparisons, alignments, and primer design.

### Sequence analysis and primer design

DNA sequences identified using the ECR Browser were imported into Vector NTI (VNTI; Invitrogen, Carlsbad, CA). Primers were selected using VNTI primer design software, and one or two restriction sites were manually added to the 5′ ends of each primer to confer additional flexibility in the cloning process and to allow constructs to be cloned into different regions of the MCS in the pFTM3GW vector (see Appendix 1 and Appendix 2). Primers from Operon Technologies (Huntsville, AL) were resuspended at 100 µM in 10 mM Tris (pH 8.0) and 1 mM EDTA.

**Figure 2:**
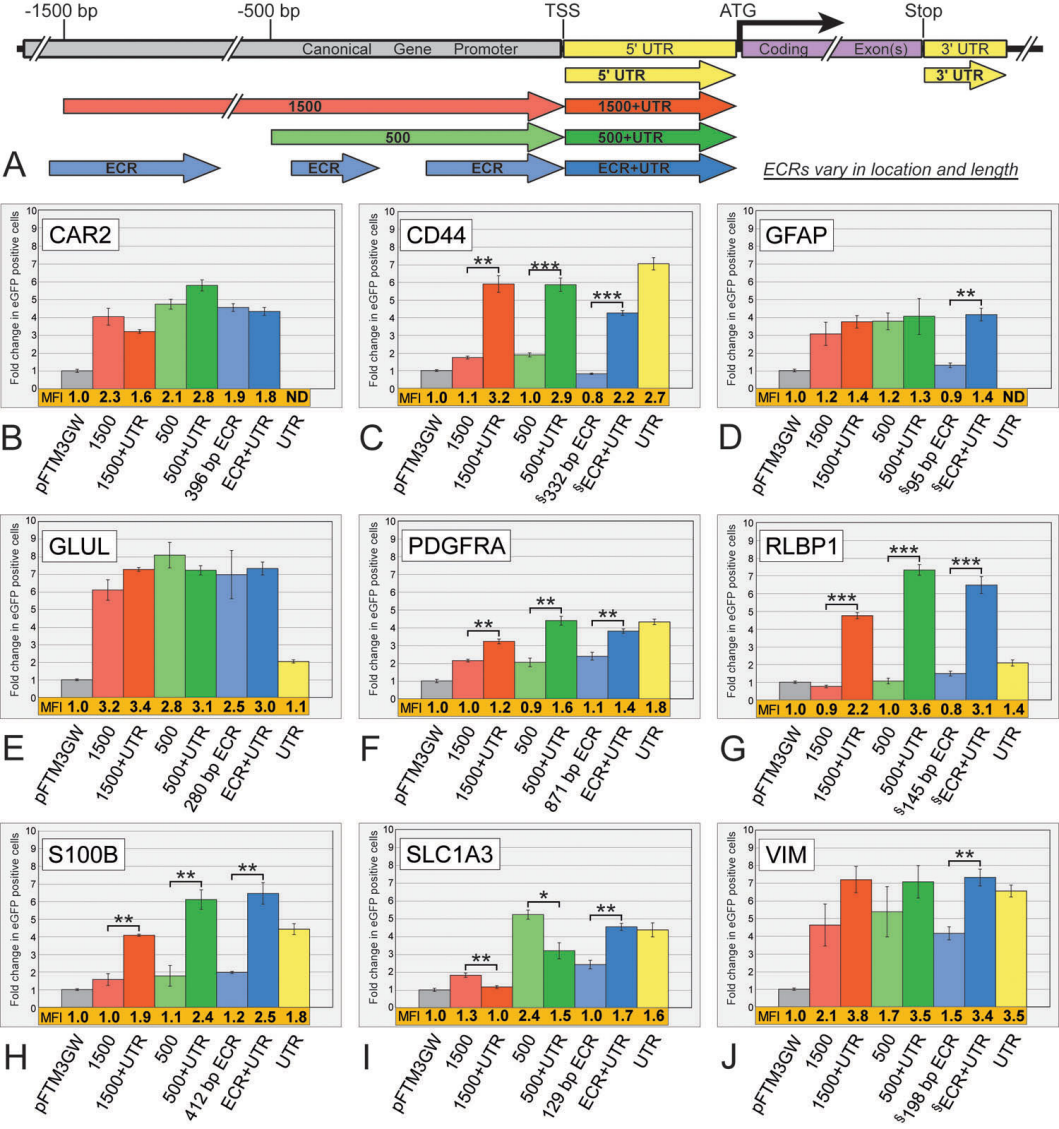
Flow cytometry analysis of promoter fragments from nine Müller cell expressed genes. **A:** Diagrammatic representation of DNA fragments analyzed here and in subsequent figures. **B-J:** Significant variability in both number of positive cells and fluorescence intensity are apparent among the 61 fragments tested. Seven fragments were analyzed for each gene: the 5′ UTR alone (yellow), a ~1500 bp (red), a ~500 bp (green), and a variable-sized evolutionarily conserved region (ECR; blue), each with and without their respective 5′ UTRs. Bars indicate fold change in number of eGFP positive cells, normalized to the promoter-less parent vector (pFTM3GW). The mean fluorescence intensity (MFI) for each construct was also normalized to the parent vector (pFTM3GW) and is shown immediately below each bar (shown in orange). UTRs for the *GFAP* (14 bp) and *CAR2* (28 bp) genes were quite short and not individually tested. Both the locationrelative to the transcriptional start site (TSS), and the size of the ECRs vary for each gene (official gene names can be found in Introduction). Refer to Appendix 1 for genomic coordinates of each construct. The symbol (§) identifies ECRs that were not immediately adjacent to the TSS (*CD44*, *GFAP*, *RLBP1*, and *VIM*), and less-conserved DNA between the ECR and TSS was included for each of these constructs. ATG is the codon for the starting methionine. Error bars represent 1 standard deviation. The single asterisk equals p<0.01, the double asterisk equals p<0.001, and the triple asterisk equals p<0.0001, using a two-tailed Student's *t*-test assuming equal variances. ND means “not determined.”

### Polymerase chain reaction amplification and cloning

Sequences were PCR-amplified from C57BL/6J mouse or Sprague-Dawley rat gDNA using 0.5 U per reaction Platinum Taq or Platinum Taq High-Fidelity Polymerases (Invitrogen). Standard reaction conditions were 2.5 mM Mg^2+^, 0.2 µM each primer, 0.2 mM deoxyribonucleotide triphosphates (dNTPs), and 10–50 ng gDNA in a 20 µl reaction volume. A list of cloned sequences, including their primer sequences and their respective chromosomal locations, are shown in Appendix 1 and Appendix 2. PCR products were typically T/A cloned into the pGemT-Easy vector (Promega, Madison, WI) and transformed into DH5alpha chemically competent cells (Invitrogen). Plasmid DNA was isolated using the QIAprep Miniprep Kit (Qiagen Corp., Valencia, CA). Each promoter fragment was digested and gel purified from pGem-T-easy, and subsequently subcloned into linearized pFTM3GW. For verification, all constructs were PCR screened, endonuclease digested, and sequenced. While most promoter sequences matched published database sequences exactly, some sequences had individual point mutations as well as small (1 or 2 bp) insertions or deletions, particularly in genomic regions containing a minisatellite repeat(s).

### Cell culture and transfection

Cell culture was performed as previously described [22] for both rat (wMCs) and human (MIO-M1) Müller cells [36]. MIO-M1 cells were a gift from University College London. NIH/3T3 (ATCC#: CRL-1658) and Neuro-2a (ATCC#: CCL-131) were obtained from the University of California, Berkeley core research facility. Briefly, approximately 200,000 Müller cells or 400,000 NIH/3T3 or Neuro-2a cells were plated per well on 12 well plates. Cells were grown in Dulbecco's Modified Eagle's Medium (DMEM) including high glucose (Invitrogen) with 10% fetal bovine serum (FBS; HyClone; Thermo Fisher Scientific, Waltham, MA) and 4 mM L-glutamine without antibiotics until they reached 90% confluence (~24 h). A transfection complex with 0.75 µg plasmid DNA (experimental or control) and 3.75 µl Lipofectamine 2000 (Invitrogen) in 200 µl of OptiMEM (Gibco; Invitrogen) medium was prepared according to the manufacturer’s specifications. Growth medium was removed and the transfection complex was added into each individual well. After 10 min, 1 ml of fresh media without antibiotics was added. Cells were cultured for approximately 40 h before flow cytometry analysis.

**Figure 3:**
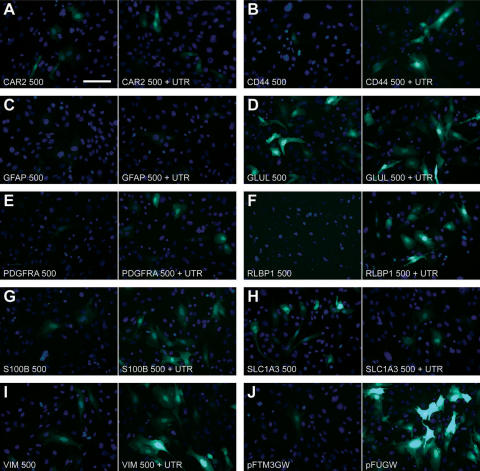
The choice of gene promoter and the presence of the gene’s 5′ untranslated region influence the level of reporter gene expression in cultured rat Müller cells. Microscopic analysis is in agreement with the flow cytometry data (Figure 2), and therefore serves as a rapid, qualitative approach to screening experimental promoters. eGFP expression is demonstrated for the 500 bp promoter fragments either with (right) or without (left) the 5′ UTR for each of the nine genes analyzed (**A-I**). Shown in (**J**) are control transfections with the backbone vector (pFTM3GW; left), or a vector containing the 1178 bp human ubiquitin-C promoter (pFUGW; right). Scale bar in (**A**) equals 10 µm.

### Flow cytometry and data analysis

Methods for flow cytometry and fluorescence microscopy were described previously [22]. Briefly, transfected wMC and MIO-M1 Müller cells were trypsinized for ~3–5 min, resuspended in 500 µl DMEM media, and maintained on wet ice. At least 20,000 cell counts were collected for each sample, and three independent samples were counted for each construct. Mean fluorescence intensity (MFI) and percent eGFP positive cells were calculated and collected for each sample under identical gating conditions within each experiment. All data were normalized to the promoter-less parent vector (pFTMGW or pFTM3GW) or pFUGW, and were subsequently plotted as relative fold-change in terms of number of eGFP labeled cells and MFIs. pFTM3GW and pFTMGW [22] employ a TB element to minimize (by 85%–90%) transgene expression by an upstream hybrid CMV/5′LTR promoter, which is necessary for virus production. The fortuitous “leakiness” of the TB element has two benefits: 1) it allows for efficient and accurate normalization across experiments; and 2) it allows for the identification of MCS-cloned elements that negatively influence transgene expression. We used Microsoft Excel for all statistical analyses, using the Student's *t*-test for all comparisons (two-sample assuming equal variances). Analyses were performed on raw data counts and collected as percent positive cells (fluorescent, flow cytometry counted). Data and errors were normalized to the promoter-less parent vectors (pFTMGW or pFTM3GW) or pFUGW. Data obtained from control plasmid transfections were set to a value of 1.0 (or 100%), which allowed for relative fold-change (increase or decrease) calculations for experimental constructs, as well as a baseline for comparison between experiments. All error bars represent (±) 1 standard deviation.

### Microscopy

For microscopic examination of eGFP expression, cells were grown on cover glass under identical conditions, and were processed as previously described [22]. Briefly, cells were transfected for approximately 24 h, rinsed with phosphate buffered saline (PBS; pH 7.4), fixed with 10% neutral buffered formalin for 15 min, rinsed again with PBS, and inverted on a microscope slide containing a drop of Vectashield (Vector Laboratories, Inc., Burlingame, CA) containing DAPI (4',6-diamidino-2-phenylindole) as a nuclear counterstain (blue). Images were collected using Zeiss Axiovision 4.4 software (Carl Zeiss, Oberkochen, Germany) with a fixed exposure time, and all images were identically post-processed with Adobe Photoshop (Adobe Systems, San Jose, CA).

**Figure 4:**
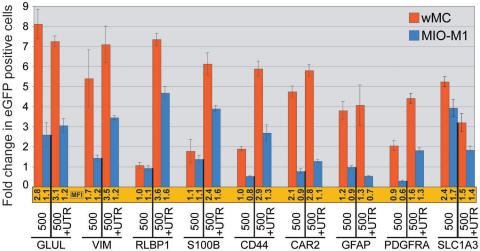
Comparison of promoter regulated gene expression in rat (wMC) and human (MIO-M1) Müller cells. Müller cells were transfected with 500 bp or 500+UTR genomic DNA constructs (mouse) from each of the nine genes analyzed. Expression was more robust in rat Müller cells (red bars) when compared with human MIO-M1 Müller cells (blue bars), although the trends in promoter-driven transgene expression are similar between species and within genes. Bars indicate fold change in number of eGFP positive cells, normalized to the promoter-less parent vector (pFTM3GW). The mean fluorescence intensity (MFI) for each construct was also normalized to the parent vector (pFTM3GW) and is shown immediately below each bar (shown in orange). Refer to Appendix 1 for genomic coordinates of each construct. Error bars represent 1 standard deviation.

## Results

### Control plasmids and normalization

The pFTM3GW LV transfer vector (Figure 1) is an improved version of pFTMGW [22] (see Methods for details). This vector utilizes a TB element to greatly reduce expression (by 85%–90%) from the hybrid hCMV/5′LTR (a promoter element required for in vitro virus production). In each of the experiments presented here, we utilize the low basal level of “read-through” expression (~10%–15%) as our baseline for normalization, except for the comparative expression in NIH/3T3 and Neuro-2a cells, for which the pFUGW plasmid (containing a portion of the human ubiquitin-C promoter) was used for normalization. Thus, the percentage of eGFP positive cells counts for pFTMGW or pFTM3GW control plasmids were assigned a value of 1.0, and all measurements of experimental constructs are shown in terms of fold change (in number of eGFP positive cells) relative to pFTMGW, pFTM3GW, or pFUGW. In addition, all MFI's were similarly normalized: these data are indicated in each graph immediately under each bar (shaded with orange). Lastly, the “leakiness” of the TB permits the identification of putative repressive regulatory elements.

### Müller cell-associated promoter testing

We systematically examined the activity of 61 DNA sequences in the promoter regions of 9 Müller cell-associated genes (Figure 2, Appendix 1). Fragments cloned from each gene are schematically represented in Figure 2A, and numbered relative to the TSS. We cloned the 500 bp and the 1500 bp sequences, both with and without the adjacent 5′ UTR, for each of the nine genes (Figure 2B-2J). In addition, ECRs were cloned with and without the 5′ UTR. In some cases (*CD44*, *GFAP*, *RLBP1*, and *VIM*; see Appendix 1), the most proximal ECR was not immediately adjacent to the TSS: when cloning these ECR+UTR fragments, additional intervening sequences possessing less than our 70% homology threshold between the ECRs and 5′ UTRs, including the TSSs, were included. Finally, individual UTR sequences for seven of the nine genes were tested alone. The UTRs for *CAR2* (28 bp) and *GFAP* (14 bp) were quite small, and not individually examined.

**Figure 5:**
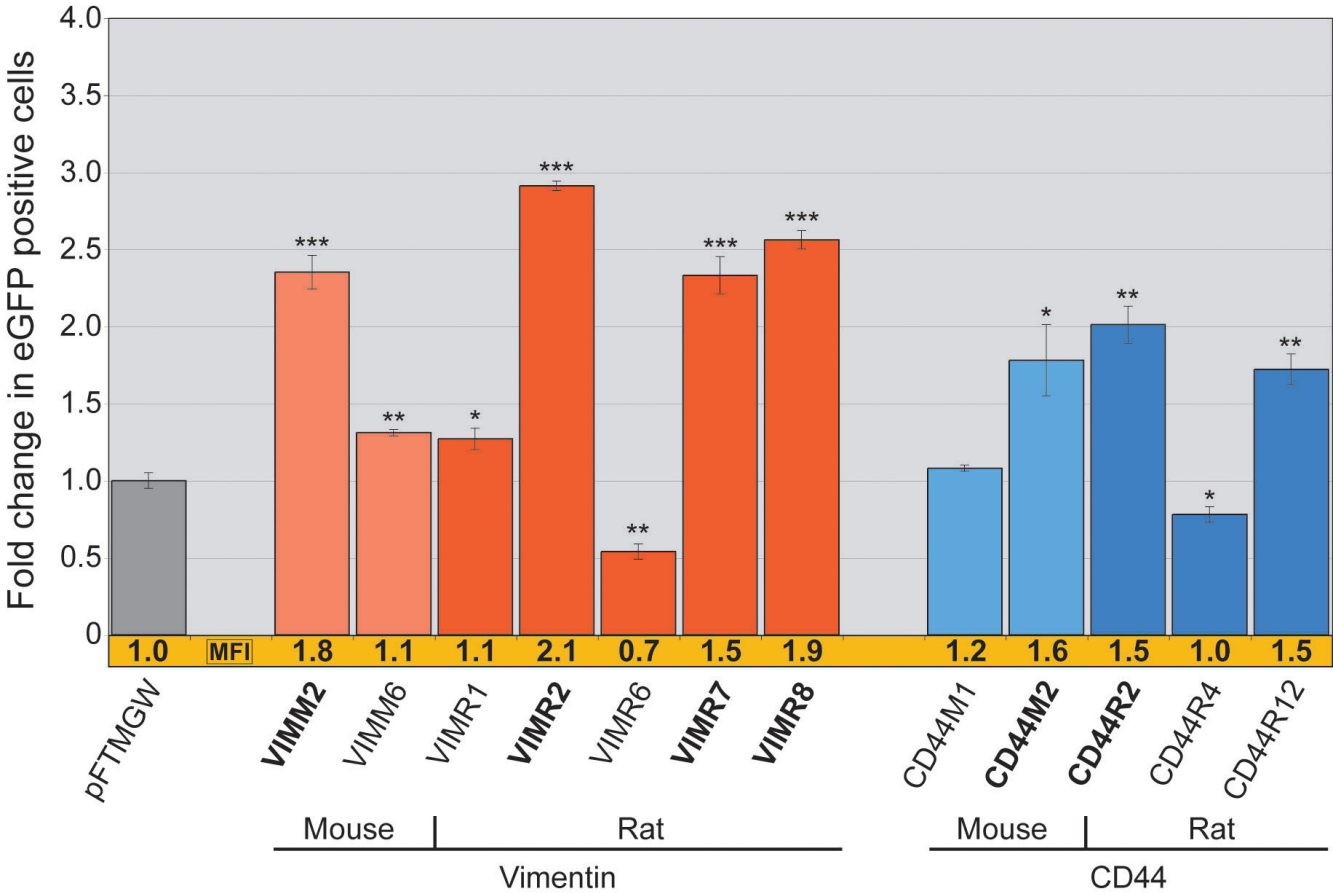
Transfection of mouse and rat evolutionarily conserved regions from the VIM and *CD44* genes in wMC (rat) Müller cells. Constructs containing either 5′ (*VIMM2*, *VIMR2*, *CD44M2*, *CD44R2*) or 3′ (VIMR7 and VIMR8) UTRs exhibited high eGFP expression levels (shown in bold). VIM constructs are shown in red, and CD44 constructs are shown in blue; lighter colors are mouse constructs and darker colors are rat constructs. Bars indicate fold change in number of eGFP positive cells, normalized to the “first-generation” promoter-less parent vector (pFTMGW). The mean fluorescence intensity (MFI) for each construct was also normalized to the parent vector (pFTMGW) [22] and is shown immediately below each bar (shown in orange). Refer to Appendix 2 for genomic coordinates of each construct. The single asterisk equals p<0.01, the double asterisk equals p<0.001, and the triple asterisk equals p<0.0001, using a two-tailed Student's *t*-test assuming equal variances. Error bars represent 1 standard deviation.

### Variable influences of the 5′ untranslated regions

In general, the UTRs alone were capable of increasing the number of eGFP positive cells two- to sevenfold (Figure 2). RLBP1 (Figure 2G) showed very low expression unless the UTR was combined with other regulatory elements (500 bp, 1500 bp, or ECR). Combining *RLBP* elements (500+UTR, 1500+UTR, or ECR+UTR) resulted in greater than four- to sevenfold more Müller cells expressing eGFP, with a concomitant increase in MFIs (up to 3.6-fold higher; 500+UTR). Similarly for the S100B gene (Figure 2H), including the UTR led to increased expression over individual constructs. For S100B, the combined constructs (500+UTR and ECR+UTR) appeared to have more of an “additive” effect on eGFP expression, whereas a “multiplicative” influence was apparent for RLBP1. The UTRs of CD44 (Figure 2С) and PDGFRA (Figure 2F) drove significant levels of eGFP expression on their own, whereas the individual 5′ elements (500 bp, 1500 bp, and ECR) resulted in considerably lower eGFP expression.

In contrast, some constructs without UTRs drove significant levels of eGFP in cultured Müller cells. For example, the 500 bp, 1500 bp, and ECR elements in CAR2 (Figure 2B), GFAP (Figure 2D; except ECR), GLUL (Figure 2E), and VIM (Figure 2J) resulted in three- to eightfold changes in the number of cells expressing eGFP. Note that the 118 bp *GLUL* UTR led to a nominal (approximately twofold) change in reporter gene expression by itself, whereas the individual constructs (with or without the UTR) resulted in six- to eightfold increases. Thus, in the case of *GLUL*, the UTR appeared to exhibit only a minor influence on gene expression, whereas upstream elements contributed significantly. Constructs isolated from the *SLC1A3* gene exhibited variable influences on eGFP expression (Figure 2I). In contrast to other genes, the *SLC1A3* 1500+UTR and 500+UTR sequences led to reduced expression relative to the 500 bp and 1500 bp fragments without the UTR (Figure 2I), suggesting a complex *cis*-regulatory network involving both positive and negative influences at the SLC1A3 promoter. Even though the cellular specificity of these regulatory elements has not been verified, it is worth noting that the short, 205 bp GFAP ECR+UTR promoter fragment drove eGFP expression as well as any other GFAP construct tested (Figure 2D).

### Microscopic examination of 500 bp elements

We screened promoter constructs using fluorescence microscopy before flow cytometry analysis, and qualitatively assessed their eGFP expression 24–40 h following transfection. Figure 3 shows fluorescent micrographs of rat Müller cells grown on cover glass and transfected with the 500 bp and 500+UTR constructs for each of the nine genes. Cultures were treated identically to those used for flow cytometry experiments, and qualitatively support the quantitative flow cytometry data shown in Figure 2. The presence of the UTR enhanced the number of eGFP-labeled cells in several experimental constructs: *CD44* (Figure 3B); *PDGFRA* (Figure 3E); *RLBP1* (Figure 3F); *S100B* (Figure 3G); and *VIM* (Figure 3I). In contrast, little or no effect was observed for *CAR2* (Figure 3A), *GFAP* (Figure 3C), or *GLUL* (Figure 3D). Lastly, the presence of the UTR in the *SLC1A3* construct (500+UTR; Figure 3H) resulted in fewer eGFP labeled cells (also see Figure 2I). Control transfections (Figure 3J) appropriately exhibited a few dim (pFTM3GW) or numerous bright (pFUGW; containing the human ubiquitin-C promoter) fluorescent wMC Müller cells.

**Figure 6:**
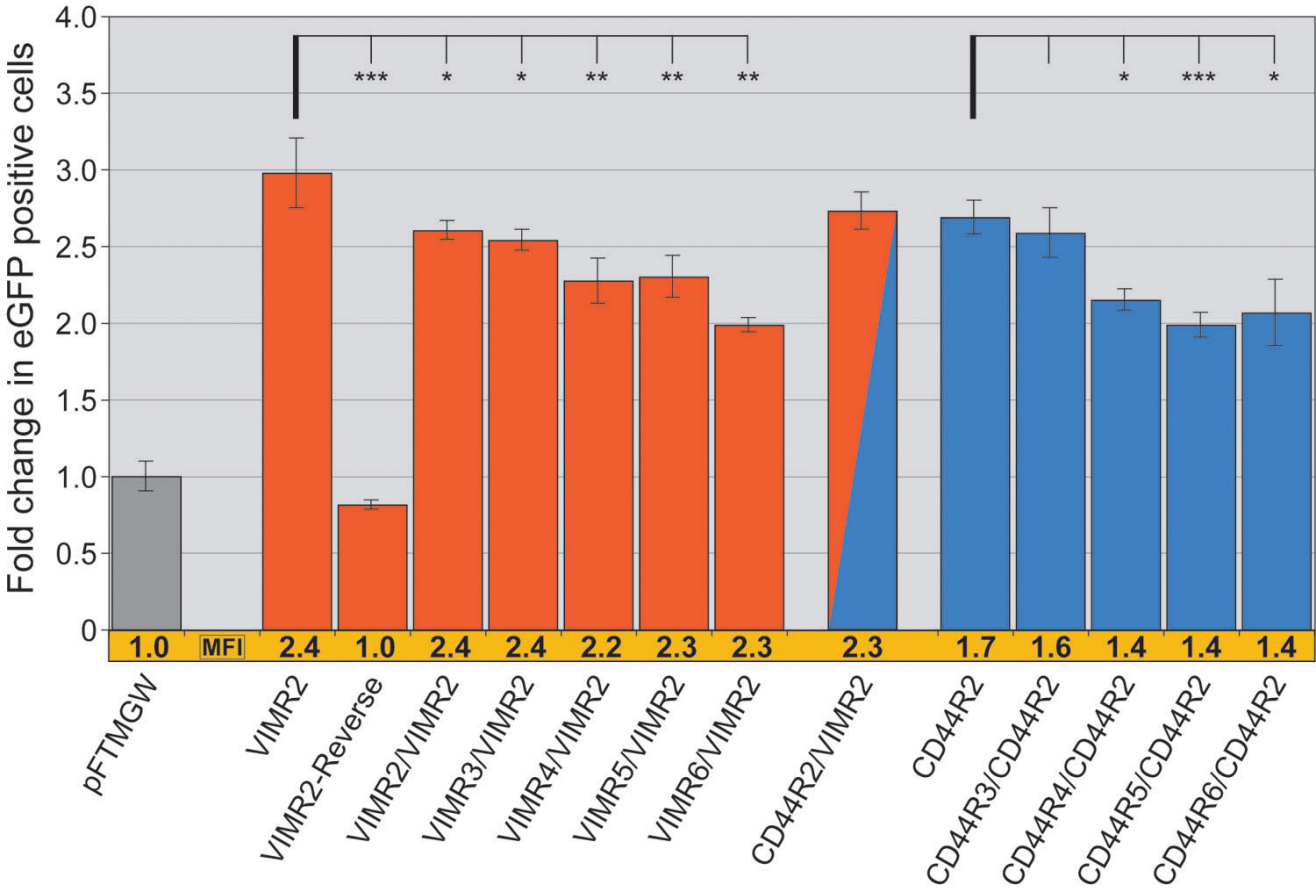
Compound promoter constructs can modulate transgene expression. Vimentin (*VIM*) and *CD44* evolutionary conserved regions (ECRs) were tested were tested in various combinations. For both genes, we examined the effect of adding gene-specific ECRs to either of the *VIMR2* or *CD44R2* element-containing vectors; in each case the added ECR was positioned 5′ to the existing cloned promoter element (*VIMR2* or *CD44R2*) within the multiple cloning site (MCS), to the, to the existing promoter element (*VIMR2* or *CD44R2*). Adding VIMR2–6 to the VIMR2-containing construct resulted in slightly lower number of cells expressing eGFP (shown in red), while the mean fluorescence intensity (MFI) remained constant. When CD44R3–6 were individually added to CD44R2 (shown in blue), both cell counts and MFIs showed a modest decrease. The VIMR2-Reverse reduced expression to near baseline levels. A chimeric promoter construct containing *VIMR2* (3′) and *CD44R2* (5′) elements (red/blue) did not change expression significantly. Bars indicate fold change in number of eGFP positive cells, normalized to the “first-generation” promoter-less parent vector (pFTMGW) [22]. The MFI for each construct was also normalized to the parent vector (pFTMGW) and is shown immediately below each bar (shown in orange). Refer to Appendix 2 for genomic coordinates of each construct. Compared to pFTMGW, all constructs (except *VIMR2R*) showed quantitatively different expression. p<0.01, using a two-tailed Student's *t*-test assuming equal variances. The asterisk equals p<0.05, the double asterisk equals p<0.01, and the triple asterisk equals p<0.001, using a two-tailed Student's *t*-test assuming equal variances. Error bars represent 1 standard deviation.

### Comparison between rat and human Müller cells

To assess orthologous promoter activity, we applied the same 500 bp and 500+UTR promoter constructs to a spontaneously generated human Müller cell line (MIO-M1 [36]). Figure 4 indicates fold changes in eGFP positive cells in both rat (red) and human (blue) cultured Müller cells. All data were normalized to the promoter-less parent plasmid, pFTM3GW (not shown). As one might expect from divergent DNA promoter sequences, the overall numbers (and MFIs) of eGFP positive human Müller cells were reduced relative to the rat (wMC) Müller cells. Importantly, though, the trends in the data were notably conserved for several genes, suggesting that some orthologous, sequence-related mechanisms are likely participating in the reporter gene expression. Some genes exhibit similar expression profiles (RLBP1, S100B, SLC1A3), while others exhibit definitive differences (CAR2 and GFAP).

### Further analysis of the mouse and rat CD44 and vimentin promoters

We computationally identified, cloned, and screened additional mouse and rat ECR sequences from the CD44 and VIM genes. A total of 32 ECRs (see Appendix 2) located both upstream and downstream (typically more distal conserved regions) of the mouse and rat CD44 and VIM coding sequences were cloned and studied by either flow cytometry or fluorescence microscopy or both. Approximately 60% (20/32) of the individual constructs resulted in expression levels that were indistinguishable from pFTMGW vector alone (see Appendix 2; constructs with “nd” in the eGFP and MFI columns). After microscopic screening (data not shown), the remaining ECRs (12/32) were analyzed by flow cytometry (Figure 5; Appendix 2). Notably, some ECRs representing the same conserved regions in mouse and rat drove eGFP expression at similar expression levels (Figure 5; *CD44M2*/*CD44R2* and *VIMM2*/*VIMR2*). Both pairs of promoter constructs resulted in roughly two- to threefold increases in number of eGFP expressing cells. Interestingly, the two rodent *VIM* genes have different TSSs; *VIMR2* (rat) is a 409 bp fragment, containing 330 bp of the *VIM* promoter and 79 bp of the VIM 5′ UTR, whereas *VIMM2* (mouse) is entirely within the *VIM* 5′ UTR. It is also important to notice the reduction in the number of eGFP positive cells (and MFI) by the *VIMR6* and CD44R4 constructs. In particular, the *VIMR6* fragment negatively influenced eGFP expression by both flow cytometry (Figure 5) and fluorescence microscopy (data not shown). Moreover, when added to the *VIMR2* promoter fragment, VIMR6 reduced the number of wMC Müller cells by ~33% (Figure 6).

**Figure 7:**
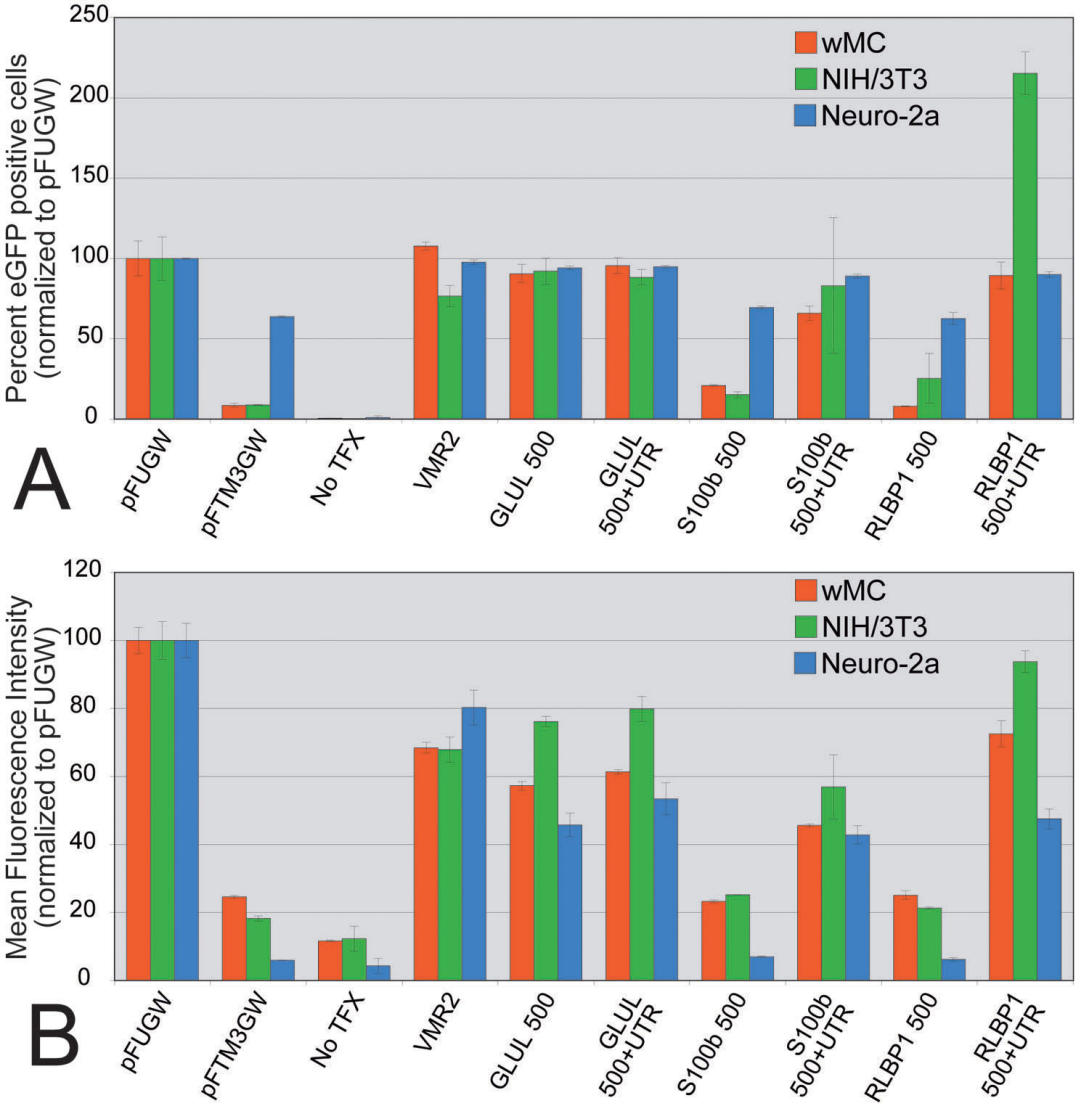
Influence of cell type on promoter-directed reporter gene expression. Promoter constructs were transfected into wMC (rat Müller cells; red), NIH/3T3 (mouse fibroblasts; green), or Neuro-2a (mouse neuroblastoma; blue). All values were normalized to pFUGW and plotted according to percent enhanced green fluorescent protein (eGFP) positive cells (**A**) or mean fluorescence intensities (MFI; **B**). **A:** Percent eGFP expressing cells are plotted for control (pFUGW, pFTMGW, and VMR2) and xperimental (*GLUL* 500 ± untranslated region (UTR), *S100B* 500±UTR, and *RLBP1* 500±UTR) constructs. Note that transfection efficiency of Neuro-2a cells was significantly higher overall (95% positive cells versus ~20% for wMC and NIH/3T3), which, consequently, resulted in significantly higher expression in the pFTM3GW control. Nevertheless, when normalized to pFUGW, cell type had little influence on the relative percentage of cells that express eGFP, with the exception of a doubling in the number of NIH/3T3 cells transfected with the RLBP1 500+UTR construct. **B:** Though absolute eGFP expression levels were quite different for the three cell types examined, similar results to those in (**A**) were obtained when the MFIs were normalized to pFUGW. Perhaps an overall trend is noticeable; NIH/3T3 cells (green) tended to offer slightly higher relative eGFP expression, and Neuro-2a cells (blue) tended to exhibit slightly lower eGFP expression than that observed for wMC Müller cells (red). No TFX indicates no transfection. Error bars represent 1 standard deviation.

### Complex and chimeric promoters are readily testable using pFTMGW or pFTM3GW

We examined the feasibility of cloning and testing in silico-designed, conjoined promoter constructs (Figure 6). We added secondary ECRs to two promising plasmid constructs containing *VIMR2* or *CD44R2* (both of rat origin). At least for *VIMR2* and *CD44R2*, the inclusion of additional ECR elements upstream of existing elements (having already been cloned into the MCS) resulted in a reduction in the number of eGFP expressing cells. The chimeric construct, *CD44R2* and *VIMR2* (red-blue bar), was not significantly different from either individual construct with respect to number of eGFP positive cells or MFIs. Duplicating the *VIMR2* element slightly decreased the number of cells, but did not affect the level of expression (MFI) when compared to the single *VIMR2* construct. Directional selectivity/specificity of the *VIMR2* promoter was confirmed by testing a construct in which *VIMR2* was cloned in the opposite orientation (VIMR2-Reverse; 3′-5′ orientation). The *VIMR2*-Reverse construct resulted in slightly fewer eGFP positive cells (p=.033) when compared to the vector alone (pFTMGW).

We also identified a short fragment from the rat vimentin gene that appeared to negatively modulate transgene expression when tested individually and when included in combination with other putative regulatory elements. The VIMR6 sequence lies ~2.8 kb upstream (5′) of the gene’s TSS, and its specific function remains to be determined. Shown in Figure 5, the 360 bp *VIMR6* ECR fragment decreased the amount of pFTMGW baseline expression by 45%, suggesting that *VIMR6* represents a putative inhibitory *cis*-regulatory element. In Figure 6, we show that combining *VIMR6* with *VIMR2* decreased the number of eGFP positive Müller cells by nearly 30% (compared to VIMR2-only levels), adding further evidence that *VIMR6* has the capacity to mitigate gene expression. However, note that VIMR6 did not decrease MFI levels. For the *CD44* gene, the CD44R4 construct exhibited similar negative-modulating characteristics as VIMR6.

### Comparison of expression in two non-glial cell types

Six experimental constructs (*GLUL* 500±UTR, *RLBP1* 500±UTR, and *S100b* 500±UTR) were tested by transfection in 3 cell types: wMC Muller cells, NIH/3T3 fibroblasts, and Neuro-2a neuroblastoma cells (Figure 7). Both cell count and MFI values for all constructs were normalized to pFUGW expression levels. Under these culture conditions it is clear that the promoter constructs tested do not restrict expression to “glial” cells. After normalization to the pFUGW construct, comparable percentages of cells (Figure 7A) and MFIs (Figure 7B) were observed for wMC, NIH/3T3, and Neuro-2a cells. Overall eGFP positive cell counts indicated that Neuro-2a cells were significantly more receptive to transfection, approaching 95% transfection efficiency (compared to ~20% for both wMC and NIH/3T3 cells, using an identical transfection protocol; data not shown). Most notably, a distinct difference was observed when NIH/3T3 cells were transfected with the *RLBP1* 500+UTR construct, which resulted in over twice as many cells becoming eGFP positive, relative to the pFUGW control (Figure 7A). In addition, it is noteworthy to point out that overall MFI levels varied significantly for the three cell types: the average eGFP intensities (arbitrary units) for the pFUGW constructs were 53.7, 9.7, and 86.5 for wMC, NIH/3T3, and Neuro-2a cells, respectively. Thus, overall strength of transgene expression appeared to be determined by the combination of promoter activity and constitutive (undetermined) cellular qualities. Nevertheless, when normalized to pFUGW, the trend with the experimental constructs was that wMC cells expressed the eGFP reporter molecule at intermediate levels, generally between that of NIH/3T3 and Neuro-2a cells.

## Discussion

We believe that endogenous delivery of neuroprotective molecules by Müller glia, by way of viral-mediated gene transfer, holds significant promise for slowing the progression of inherited retinal disease [37–39]. Neurotrophic factor delivery is a broad, mutation-independent approach to slowing the progression of neuronal cell loss in retinal damage and disease [7,40,41]. An important consideration when delivering genetically encoded molecules is how to properly regulate and control their expression once delivered to the nucleus of a targeted cell.

In an attempt to identify and initially characterize functionally active regulatory elements capable of driving transgene expression in Müller cells, we cloned promoter constructs from nine Müller cell-associated genes into our modified LV transfer vector, pFTM3GW (Figure 1). Our data suggest that flow cytometry-based analysis of computationally identified regulatory sequences is an effective first step toward identifying small, gene-specific promoters. Our data suggest that computationally identified short ECR elements have the capacity to direct robust gene expression, and that genetically diverse UTRs differentially contribute to transgene expression. Finally, our data indicate that combinatorial cloning of promoter fragments (from one or more genes) may be exploited to facilitate fine-tuning of therapeutic gene expression.

The RLBP1 and GLUL promoters exhibit robust gene expression in our transfection assays. However, these two genes, as an example, differ significantly with respect to the influence of their respective UTRs. Only when the UTR is combined with 5′ sequences (UTR+500, UTR+1500, or UTR+ECR) do the RLBP1 constructs drive significant levels of eGFP expression (Figure 2G). The number of eGFP expressing cells and the MFIs of the combination constructs are higher than the sum of the expression levels of the individual elements. In contrast, the GLUL constructs were minimally influenced by the inclusion of the UTR (only slightly higher MFIs with the UTR; Figure 2E), and all promoter constructs (with or without the associated UTR, including a short 280 bp ECR) resulted in a high number of eGFP positive cells. From these and other data, we conclude that proximal regulatory and 5′ UTR sequences in and around the gene’s “core promoter” likely operate in a gene-context specific manner and have the capacity to function as more than generic transcription initiation zones. Of course, it remains to be determined how cell type specificity (of gene transcription) is influenced by “promoter” shortening. We have shown previously that shortening a promoter does not, a priori, sacrifice gene expression specificity [22]. However, the experiments presented here (Figure 7) suggest that, at least in culture, glial-associated promoter elements do have the capacity to express equally well in non-glial cell types, such as NIH/3T3 fibroblasts and Neuro-2a neuroblastoma cells.

Analysis of *CD44* and *VIM* ECRs indicates that promoter activity can be modulated. Our data suggest that duplicating (*VIMR2*/*VIMR2*) or combining (*CD44R2*/*VIMR2*) active elements in the same construct does not result in higher transgene expression, as we had originally hypothesized. On the contrary, adding ECRs to functional promoter elements (*VIMR2* and *CD44R2*) appeared to reduce reporter gene expression when compared to the single-element constructs (Figure 6). In the case of the *VIMR2*/*VIMR2* construct, duplicating the same element actually reduced expression by ~15%, suggesting a possible competition for transcription factor binding sites. It seems reasonable to hypothesize that providing additional spacing between promoter elements (including small “stuffer” sequences) may improve accessibility of transcription factors, but this has yet to be tested. It will be important to determine if individual ECRs can be combined to increase transgene expression in future studies.

Including the VIMR6 element in a combination construct (*VIMR6*/*VIMR2*; Figure 6) also reduced reporter gene expression, similar to the influence of this element on the empty vector (Figure 5). These data suggest that VIMR6 may function as a “partial repressor” and could conceivably be included in future constructs to reduce expression levels of active promoters. Importantly, reversing the orientation of the *VMR2* construct (*VIMR2*-Reverse; Figure 6) failed to drive gene expression, reaffirming the expectation that promoter orientation is critical for directed gene expression. Lastly, and interestingly, 3′ UTR constructs from the VIM gene (*VIMR7* and *VIMR8*) drove eGFP expression nearly as well as the 5′ UTR (VIMR2). Future experiments will test additional combinations of ECRs, 5′ UTRs, and 3′ UTRs from other genes, as well as the resultant cellular specificity of such constructs. Thus, we believe that pFTM3GW is a powerful tool that enhances our ability to examine multiple regulatory elements in single promoter constructs, which will enable detailed testing and analysis of in silico and in vitro engineered regulatory constructs.

We have shown previously [22] that quantification of eGFP using flow cytometry is as accurate as quantitative RT–PCR analysis. Moreover, flow cytometry analysis of transfected cells is faster, cheaper, and less labor intensive than quantitative RT–PCR, and therefore improves quantitative throughput and analysis of promoter-regulated gene expression. Importantly, though, microscopic screening of transfected cells is a very rapid and reliable method to quickly and qualitatively assess promoter strength (see Figure 3). Our transfection data suggest that 1) both 5′ and 3′ UTRs can impart generally positive influences on gene expression; 2) short DNA sequences (500 bp or less) can drive expression levels as robustly as the longer, canonical promoters; and 3) combinations of regulatory elements can measurably influence promoter activity. Future experiments will focus on characterizing promoter cell-specificity and assessing the concordance of expression levels between in vitro and in vivo experimental assays.

## Appendix 1. Full-length and proximal promoter fluorescence data, genomic fragment locations, and primer sequences.

To access the data, click or select the words “Appendix 1.” We identified and cloned the full-length promoters (~1500 bp), proximal promoter regions (~500 bp), the most proximal evolutionarily conserved regions (ECRs) to the transcription start site for each of nine genes identified in the literature as being potential or known Müller cell markers: *CAR2*, *CD44*, *CRALBP*, *GFAP*, *GLUL*, *PDGFRA*, *S100B*, *SLC1A3*, and *VIM*. The sequences were cloned either including or excluding the UTR, and then evaluated with fluorescent microscopy and flow cytometry. The table shows the eGFP expression and the mean fluorescent intensity, with values normalized and expressed as fold change relative to pFTM3GW. Restriction endonuclease is abbreviated REN. The “†” symbol identifies small UTRs that were not individually tested. The “‡” symbol denotes very large UTRs; only the ~200 bp regions immediately following the TSS were analyzed. eGFP and MFI values are shown as fold change relative to pFTM3GW vector alone. An (R) denotes that gene is arranged in a “reversed” orientation (3′-5′) relative to the chromosome’s centromere. The “§” symbol denotes ECR not located immediately proximal to the TSS, and some intervening sequence was included in ECR+UTR constructs. “NM”numbers immediately below the gene acronym refer to RefSeq mRNA ID numbers.

## Appendix 2. Additional evolutionarily conserved region fluorescence data, genomic fragment locations, and primer sequences.

To access the data, click or select the words “Appendix 2.” Additional evolutionarily conserved regions (ECR) from *CD44* and vimentin (*VIM*) were further studied; 32 sequences were PCR amplified from C57BL/6J mouse and Sprague-Dawley rat genomic DNAs, cloned into the pFTMGW vector, and transfected into cultured rat Müller cells. The sequences were initially screened by fluorescence microscopy. Subsequently, eGFP positive constructs were analyzed by flow cytometry. The table shows the eGFP expression and the mean fluorescent intensity (MFI), with values normalized and expressed as fold change relative to pFTMGW alone. Restriction endonuclease is abbreviated REN. The “†” symbol identifies referenced in Geller et al. (2007) [22] as Vim409. The “‡” (nd) denotes flow cytometry was not performed on constructs showing eGFP expression qualitatively similar to vector (pFTMGW) alone. eGFP and MFI values are shown as fold change in expression relative to pFTMGW vector alone. An (R) denotes that gene is arranged in a “reversed” orientation (3′-5′) relative to the chromosome’s centromere. The “#” symbol represents that tested in combination with *VIMR2* or *CD44R2*; Figure 6. The “^” symbol denotes cloned and tested in reverse orientation; Figure 6. The “@” denotes sequence includes the 5′ UTR, the first protein coding exon, and part of the first intron. “NM” numbers immediately below the gene acronym refer to RefSeq mRNA ID numbers.
